# Association between cardiometabolic index and biological aging in the US population: evidence from NHANES 2015–2020

**DOI:** 10.3389/fnagi.2024.1507035

**Published:** 2024-11-29

**Authors:** Miao Sun, Shuang Bao

**Affiliations:** Department of Neurology, General Hospital of Northern Theater Command, Shenyang, China

**Keywords:** cardiometabolic index, biological aging, phenotypic age, NHANES, chronological age

## Abstract

**Purpose:**

It is crucial to identify biomarkers that influence the aging process and associated health risks, given the growing severity of the global population aging issue. The objectives of our research were to evaluate cardiac metabolic index (CMI) as a novel biomarker for identifying individuals at increased risk of accelerated biological aging and to assess its use in guiding preventive strategies for aging-related health risks.

**Methods:**

The National Health and Nutrition Examination Survey (NHANES) provided cross-sectional data on participants with complete information on CMI, phenotypic age (PA), and other variables. Analyses of variance and weighted χ^2^ tests were conducted to assess differences between groups. The relationship between CMI and biological aging was investigated using a weighted multivariate logistic regression model, restricted cubic spline (RCS) regression analysis, subgroup analysis, and interaction testing.

**Results:**

A positive correlation between CMI and biological aging was observed in 6,272 participants. RCS regression analysis confirmed the non-linear relationship, identifying significant inflection point at 1.10. In the crude or adjusted models, the OR (95% CI), for the highest group versus the reference were 3.608 (3.108, 4.188), 3.397 (2.920, 3.952), and 1.550 (1.299, 1.850), respectively, when categorizing CMI into different groups. Subgroup analyses and interaction tests indicate that the association between CMI and biological aging remained consistent across different subgroups. Gender, race, education level, marital status, poverty income ratio (PIR), drinking status and diabetes had an interaction with CMI in relation to biological aging.

**Conclusion:**

An elevated CMI is linked to increased risk for biological aging. This relationship may inform more effective prevention and treatment strategies for biological aging in the future. CMI be integrated into routine health screenings or aging assessments by healthcare professionals.

## 1 Introduction

With one-fifth of the world’s population predicted to be 65 or older by 2030, population aging is a global problem (Rudnicka et al., 2020). A steady decrease of physiological function is a hallmark of aging. It is believed to result from a build-up of molecular alterations or “hallmarks” that impair tissues’ and organs’ ability to function and recovery (Chakravarti et al., 2021; López-Otín et al., 2023). This, in turn, is thought to cause chronic morbidities, such as metabolic, cardiovascular, neoplastic, and neurodegenerative disorders, as well as geriatric symptoms like frailty and immobility (Abbasi et al., 2023; Wagner et al., 2023; Zhou et al., 2023; Iskusnykh et al., 2024; Montégut et al., 2024). An innate biological process that is adaptable and responsive to therapeutic interventions coexists with aging. Using of various genetic, nutritional, and pharmaceutical interventions, scientists have made impressive strides in the last few decades in extending the lifespan (Mkrtchyan et al., 2020; Sourada and Kuglík, 2020; Wang et al., 2022). Therefore, it is crucial to identify biomarkers that influence the aging process and associated health risks, given the growing severity of the global population aging issue. To uncover new insights into the management and delay of the aging process, this study intends to investigate possible associations between PA, a crucial marker of biological aging, and CMI.

PA is a crucial idea connected to biological aging (Liu et al., 2018; Kuo et al., 2021). Generally, chronological age (CA) and clinical biomarkers, and blood cell parameters are utilized to evaluate PA. Given that PA provides a more accurate representation of how the body ages than CA, studies have indicated that PA is a good predictor of death, chronic morbidities, and a decline in physical function (Kuo et al., 2022). Genetic predispositions and poor lifestyle choices, like heavy smoking, excessive alcohol use, chronic illnesses, and cancer, all contribute to an increased PA. On the other hand, living a healthy lifestyle that includes eating fruits and vegetables and engaging in moderate exercise might reduce PA (Noren Hooten et al., 2022; Li et al., 2024a; Wu et al., 2024).

CMI was introduced as a novel metric by Wakabayashi and Daimon (2015) to evaluate visceral obesity using blood lipid markers and the weight-to-height ratio (WHtR). WHtR, a measure of abdominal obesity that makes more sense than just measuring waist circumference (WC). It has been shown that WC or body mass index (BMI) as cardiovascular disease risk factors are less reliable discriminators than WHtR. Because BMI measurements do not distinguish between trunk and visceral obesity, whereas anatomical fat distribution is considered important because it produces different metabolic effects (Chen R. et al., 2022; Tao et al., 2024). However, CMI simultaneously takes into account triglyceride (TG) and high-density lipoprotein cholesterol (HDL-C), which are crucial indicators of cardiovascular risk and obesity (Liu C. et al., 2022; Baratta et al., 2023; Nussbaumerova and Rosolova, 2023). Survies indicate that the CMI is connected to cardiovascular illnesses, metabolic syndrome, and other conditions, implying the importance of it for linked disease screening (Lazzer et al., 2023; Miao et al., 2023; Sun et al., 2023; Ye et al., 2024). According to recent studies, people with high CMI may have more systemic inflammation (Carvalho et al., 2024; Xu B. et al., 2024). Conversely, regular exercise is linked to a large reduction in CMI (Xue et al., 2024). Moreover, elevated CMI is significantly correlated with insulin resistance (Feng et al., 2024; Song et al., 2024; Wu and Xu, 2024). However, physical activity, insulin resistance and inflammation are intimately associated with aging (Kurauti et al., 2021; Abbasi et al., 2023; Butt et al., 2024; Singh et al., 2024). Additionally, aging is significantly impacted by BMI (Etzel et al., 2022; Lundgren et al., 2022).

To our knowledge, no previous research has examined the relationship between biological aging and CMI. Thus, the objectives of our research were to assess the correlation between biological aging and CMI, to offer guidance on the prevention and management of aging.

## 2 Materials and methods

### 2.1 Data source

The database employed in this analysis, a longitudinal cohort study, was provided by the NHANES database, a nationally representative database that collects significant data on the health of the American public. By using a multistage, stratified random sampling approach, NHANES guarantees that a national sample is represented. A total of 34,785 participants’ data were discovered after we screened and analyzed data from 2015 to 2020. The National Center for Health Statistics’ Research Ethics Review Board thoroughly examined and approved the study involving human subjects, and each participant gave signed agreements indicating their informed consent.

### 2.2 Study participants

**FIGURE 1:**
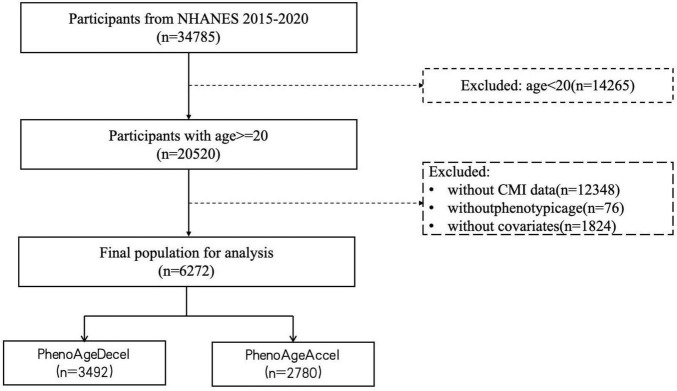
The flow chart of participant selection.

Using the following exclusion criteria, the analytical sample was reduced to 6,272 subjects: (1) individuals under the age of 20 years; (2) individuals lacking a complete CMI value; (3) individuals lacking a phenotypic age value; (4) individuals lacking records of necessary covariates, such as gender, age, race, education level, marital status, PIR, smoking, drinking, physical activity, BMI, the history of diabetes, hypertension, heart failure, stroke, and cancer. Figure 1 illustrates the inclusion and exclusion standards.

### 2.3 Assessment of CMI

As previously mentioned, anthropometric and biochemical data, such as height, WC, TG and HDL-C were used to compute CMI. The units used were milligrams per deciliter (mg/dl) for HDL-C and TG, and centimeters (cm) for height and WC. The CMI was calculated using the following formula (Liu et al., 2021):


$$\text{CMI}=\frac{\text{TG}}{\mathrm{HDL}-C}\times \frac{\text{WC}}{\text{height}}$$

For the purposes of our study, CMI was regarded as a continuous exposure variable, and all recruited participants were stratified into quartiles with cut-off values for subsequent analyses: Q1 group (CMI ≤ 0.59), Q2 group (0.60 ≤ CMI ≤ 1.06), Q3 group (1.07 ≤ CMI ≤ 1.92), and Q4 group (CMI ≥ 1.93).

### 2.4 Assessment of PA

CA and nine biomarkers—albumin, creatinine, glucose, C-reactive protein (CRP), lymphocyte percentage, mean cell volume, erythrocyte distribution width, alkaline phosphatase, and white blood cell count—were used to calculate the PA. This is a metric for the expected age in a population that is correlated with the predicted risk of death for an individual. This indicator is widely used in the literature to identify risk factors for morbidity and mortality, to assess the effectiveness of treatments, and to elucidate the aging process (Levine et al., 2018; Chen L. et al., 2022). PA was determined using the formula (Liu W. et al., 2024):


$$\,\mathrm{Phenotypic}\,\mathrm{age}=\,\,141.50+\frac{\mathrm{Ln}\,\left[-0.00553\times \mathrm{Ln}\,\left(\exp\left(\frac{-1.51714\times \text{exp}\,\left(\mathrm{xb}\right)}{0.0076927}\right)\right)\right]}{0.09165}$$

xb=-19.907-0.0336×Albumin+0.0095×Creatinine   

   +0.1953×Glucose+0.0954×LnCRP-0.00120×   

   LymphocytePercent+0.0268×MeanCellVolume   

   +0.3306×RedCellDistributionWidth+0.00188   

   ×AlkalinePhosphatase+0.0554×WhiteBloodCell   

   Count+0.0804×Chronologicalage

### 2.5 Assessment of biological aging

The residual of PA, which was corrected for CA using linear regression, was used to compute phenotypic accelerated age. Individuals classified as having phenotypic accelerated aging (PhenoAgeAccel) if their accelerated age was greater than 0, and as having phenotypic decelerated aging (PhenoAgeDecel) if their accelerated age was less than 015.

### 2.6 Assessment of covariates

The current study collected critical demographic data, such as age, gender, race (Mexican American, non-Hispanic white, non-Hispanic black, other races), education (below high school, high school or equivalent, high school above), marital status (married or living with a partner, living alone), PIR [PIR: < 1.3 (low), 1.3 ≤ to ≤ 3.5 (medium), > 3.5 (high)], BMI [BMI: < 25 (normal), 25 ≤ to ≤ 30 (overweight), > 30 (obesity)], smoking status was split into three categories: former smokers (those who had smoked at least 100 cigarettes in their lifetime and were currently giving up smoking); never smokers (those who had less than 100 cigarettes in their lifetime); and current smokers (those who had at least 100 cigarettes in their lifetime and were currently smoking), fewer than 12 alcohol-based drinks in the previous year (yes, no), physical activity was split into two categories: activity partners (those who had a minimum of 150 min per week of moderate-intensity or 75 min per week of vigorous-intensity physical activity), while others were classified as inactivity partners, the history of hypertension, diabetes, stroke, heart failure, cancer also were extracted from the database (Liu H. et al., 2022; Liu H. et al., 2024).

### 2.7 Statistical analysis

R software (version 4.2.2) was used for all statistical studies. Sampling weights were utilized in all analyses to interpret the complex NHANES survey design, in accordance with the NHANES analytical standards. Mean ± standard deviation (SD) was used to express continuous variables. Frequencies and percentages were used to express the data for categorical variables. For categorical variables, a χ^2^ test was performed to compare the baseline characteristics between groups, whereas analysis of variance was employed for continuous data. The relationship between biological aging and CMI level was examined using multivariable logistic regression models. OR (Odds Ratio) values and 95% confidence interval (95% CI) were obtained from logistic regression models, which were calculated to measure the strength of association between each independent variable and the outcome. Age, gender, ethnicity, PIR, education level, marital status, BMI, smoking, alcohol status, and history of hypertension, diabetes, heart failure, stroke, and cancer were all taken into account while adjusting the multivariable logistic regression models. Three criteria were used to choose confounding variables: clinical relevance, a *P*-value in univariate analysis of less than 0.05, and the availability of enough event data to build a strong regression model. To address concerns about over-adjustment for models, we conducted sensitivity analyses to assess the robustness of Model II and examined the consistency of our main findings across different model specifications. This approach confirmed that the addition of these covariates did not significantly impact the stability or interpretability of the key associations, supporting the robustness of Model II. The nonlinear correlations between CMI and biological aging (4 nodes, with the 25th percentile serving as a reference point) were evaluated using the RCS approach. For all analyses, a significance threshold < 0.05 was considered statistically significant.

## 3 Results

### 3.1 Baseline characteristics

In total, 6,272 participants were taken into account for this investigation. The CA was 50.28 ± 17.22 years, PA was 50.84 ± 20.03 years, and 50.4% of the individuals were male. PhenoAgeDecel and PhenoAgeAccel participants showed different characteristics. Overall, older participants, males, non-Hispanic Black individuals, lower education levels, living alone, lower PIR, higher BMI, higher likelihood of smoking, greater probability of not drinking, more likely to have hypertension, diabetes, heart failure, stroke, or cancer, and those with higher CMI levels were more likely to experience PhenoAgeAccel (*p* < 0.05). The baseline features of participants were summarized in Table 1.

**TABLE 1:** Baseline characteristics of the study participants.

	Total (*n* = 6,272)	PhenoAge deceleration (*n* = 3,492)	PhenoAge acceleration (*n* = 2,780)	*P*-value
Chronological age, mean ± SD	50.28 ± 17.22	48.70 ± 17.01	52.26 ± 17.29	< 0.001
Phenotypic age, mean ± SD	50.84 ± 20.03	44.04 ± 17.32	59.39 ± 19.93	< 0.001
**Gender, *n* (%)**				< 0.001
Male	3,162 (50.4)	1,662 (47.6)	1,500 (54.0)	
Female	3,110 (49.6)	1,830 (52.4)	1,280 (46.0)	
**Race, *n* (%)**				< 0.001
Mexican American	886 (14.1)	520 (14.9)	366 (13.2)	
No-Hispanic White	2,335 (37.2)	1,336 (38.3)	999 (35.9)	
No-Hispanic Black	1,409 (22.5)	582 (16.7)	827 (29.7)	
Other Race/Ethnicity	1,642 (26.2)	1,054 (30.1)	588 (21.2)	
**Education level, *n* (%)**				< 0.001
Less than high school	1,131 (18.0)	583 (16.7)	548 (19.7)	
High school grade or equivalent	1,477 (23.5)	721 (20.6)	756 (27.2)	
College or above	3,664 (58.5)	2,188 (62.7)	1,476 (53.1)	
**Marital status, *n* (%)**				< 0.001
Married or living with a partner	3,769 (60.1)	2,186 (62.6)	1,583 (56.9)	
Living alone	2,503 (39.9)	1,306 (37.4)	1,197 (43.1)	
**PIR, *n* (%)**				< 0.001
Low	1,721 (27.4)	832 (23.8)	889 (32.0)	
Medium	2,558 (40.8)	1,351 (38.7)	1,207 (43.4)	
High	1,993 (31.8)	1,309 (37.5)	684 (24.6)	
**Smoker status, *n* (%)**				< 0.001
Former	2,018 (32.2)	1,040 (29.8)	978 (35.2)	
Never	3,358 (53.5)	2,109 (60.4)	1,249 (44.9)	
Current	896 (14.3)	343 (9.8)	553 (19.9)	
**Alcohol status, *n* (%)**				< 0.001
Non-drinkers	2,745 (43.8)	1,409 (40.3)	1,336 (48.1)	
Drinkers	3,527 (56.2)	2,083 (59.7)	1,444 (51.9)	
**Physical activity, *n* (%)**				< 0.001
Inactivity	3,310 (52.8)	1,753 (50.2)	1,557 (56.0)	
Activity	2,962 (47.2)	1,739 (49.8)	1,223 (44.0)	
**BMI, *n* (%)**				< 0.001
Normal	1,628 (26.0)	1,213 (34.7)	415 (14.9)	
Overweight	1,997 (31.8)	1,263 (36.2)	734 (26.4)	
Obese	2,647 (42.2)	1,016 (29.1)	1,631 (58.7)	
**Hypertension, *n* (%)**				< 0.001
No	3,926 (62.6)	2,494 (71.4)	1,432 (51.5)	
Yes	2,346 (37.4)	998 (28.6)	1,348 (48.5)	
**Diabetes, *n* (%)**				< 0.001
No	5,117 (81.6)	3,180 (91.1)	1,937 (69.7)	
Borderline	168 (2.7)	71 (2.0)	97 (3.5)	
Yes	987 (15.7)	241 (6.9)	746 (26.8)	
**Heart failure, *n* (%)**				< 0.001
No	6,046 (96.4)	3,442 (98.6)	2,604 (93.7)	
Yes	226 (3.6)	50 (1.4)	176 (6.3)	
**Stroke, *n* (%)**				< 0.001
No	6,002 (95.7)	3,400 (97.4)	2,602 (93.6)	
Yes	270 (4.3)	92 (2.6)	178 (6.4)	
**Cancer, *n* (%)**				0.002
No	5,642 (90.0)	3,178 (91.0)	2,464 (88.6)	
Yes	630 (10.0)	314 (9.0)	316 (11.4)	
**CMI, mean ± SD**	1.56 ± 1.91	1.27 ± 1.33	1.93 ± 2.39	< 0.001

PIR, poverty income ratio; BMI, body mass index; CMI, cardiometabolic index.

### 3.2 Association of CMI levels and biological aging

**FIGURE 2:**
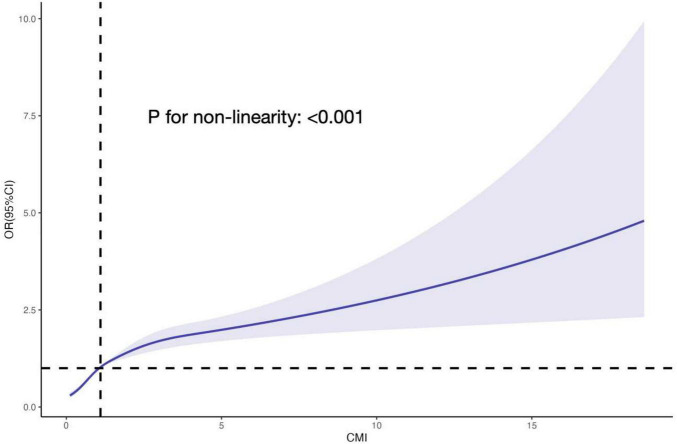
The RCS curve of the association between CMI and PhenoAgeAccel odds ratio among all the study participants. The associations between CMI and biological aging with inflection point at 1.10 was discovered after multivariable adjustment. *P*-values for non-linear were *P* < 0.001. RCS, restricted cubic spline; CMI, cardiometabolic index; OR, odds ratio.

The relationship between CMI level and biological aging was examined using weighted multivariable logistic regression models. The participants were categorized into quartiles of CMI for stratification purposes. The OR (95% CIs) for the highest group versus the reference (the lowest group) were 3.608 (3.108, 4.188), 3.397 (2.920, 3.952), and 1.550 (1.299, 1.850) for the unadjusted model, model I (adjusting for gender, year), and model II (adjusting for gender, age, race, education level, marital status, PIR, smoke, alcohol, physical activity, BMI, diagnosis of hypertension, diabetes, heart failure, stroke, and cancer), respectively, when categorizing CMI into different groups. Both the unadjusted and adjusted models showed a significant rise in the incidence of biological aging as the CMI increased. *P*-values for the trend were *P* < 0.001 (Table 2). Furthermore, we examined the dose response connection between the CMI and biological aging using limited Cubic Splines. The associations between CMI and biological aging with inflection points at 1.10 was discovered after multivariable adjustment. *P*-values for non-linear were *P* < 0.001 (Figure 2).

**TABLE 2:** Association between CMI and PhenoAgeAccel in multiple logistic regression analyses model.

CMI categorical	Crude model		Model I		Model II	
	**OR (95% CI)**	***P*-value**	**OR (95% CI)**	***P*-value**	**OR (95% CI)**	***P*-value**
Q1 (≤ 0.59)	Ref		Ref		Ref	
Q2 (0.60–1.06)	1.804 (1.554, 2.094)	< 0.001	1.736 (1.493, 2.018)	< 0.001	1.197 (1.014, 1.413)	0.034
Q3 (1.07–1.92)	2.601 (2.243, 3.017)	< 0.001	2.480 (2.135, 2.880)	< 0.001	1.359 (1.145, 1.612)	< 0.001
Q4 (≥ 1.93)	3.608 (3.108, 4.188)	< 0.001	3.397 (2.920, 3.952)	< 0.001	1.550 (1.299, 1.850)	< 0.001
*P* for trend	< 0.001		< 0.001		< 0.001	

CMI, cardiometabolic index; OR, odds ratio; CI, confidence interval; Ref, reference; Crude model adjust for: None; Model I adjust for: Gender; Age; Model II adjust for: Gender; Age; Race; Education level; Marital status; Poverty income ratio; Smoke; Alcohol; Physical activity; Body mass index; Hypertension; Diabetes; Heart failure; Stroke; Cancer.

### 3.3 Stratified analyses

The following variables were analyzed using stratified analyses: gender, age, race, education level, marital status, PIR, smoking, drinking status, physical activity, BMI, and diagnoses of hypertension, diabetes, heart failure, stroke and cancer. As shown in Table 3, except for those with borderline diabetes and heart failure, a higher CMI level was linked to an increased risk of biological aging in most subgroups. Notably, gender, race, education level, marital status, PIR, drinking status and diabetes had an interaction with CMI in relation to biological aging. The correlation between CMI and biological aging was more significant in female (OR: 1.51; 95% CI: 1.41–1.62), other race (OR: 1.51; 95% CI: 1.39–1.64), high education (OR: 1.39; 95% CI: 1.31–1.47), living alone (OR: 1.41; 95% CI: 1.32–1.52), high PIR (OR: 1.37; 95% CI: 1.28–1.48), non-drinkers (OR: 1.40; 95% CI: 1.31–1.50), and diabetes groups (OR: 1.53; 95% CI: 1.34–1.76).

**TABLE 3:** Subgroup analysis by CMI.

Subgroup	No. of PhenoAge acceleration	OR (95% CI)	*P*-value	*P* for interaction
**Gender**				< 0.001
Male	1,500	1.22 (1.16, 1.28)	< 0.001	
Female	1,280	1.51 (1.41, 1.62)	< 0.001	
**Age, year**				0.266
≤ 40	790	1.36 (1.27, 1.46)	< 0.001	
> 40	1,990	1.29 (1.23, 1.36)	< 0.001	
**Race**				0.0283
Mexican American	366	1.19 (1.11, 1.30)	< 0.001	
No-Hispanic White	999	1.47 (1.37, 1.58)	< 0.001	
No-Hispanic Black	827	1.46 (1.28, 1.68)	< 0.001	
Other	588	1.51 (1.39, 1.64)	< 0.001	
**Education level**				< 0.001
Less than high school	548	1.19 (1.10, 1.28)	< 0.001	
High school grade or equivalent	756	1.30 (1.19, 1.42)	< 0.001	
College or above	1,476	1.39 (1.31, 1.47)	< 0.001	
**Marital status**				0.0426
Married or living with a partner	1,583	1.29 (1.23, 1.36)	< 0.001	
Living alone	1,197	1.41 (1.32, 1.52)	< 0.001	
**PIR**				0.0101
Low	889	1.21 (1.13, 1.30)	< 0.001	
Medium	1,207	1.35 (1.27, 1.44)	< 0.001	
High	684	1.37 (1.28, 1.48)	< 0.001	
**Smoker status**				0.624
Former	978	1.31 (1.23, 1.40)	< 0.001	
Never	1,249	1.31 (1.24, 1.39)	< 0.001	
Current	553	1.30 (1.16, 1.47)	< 0.001	
**Alcohol status**				0.0165
Drinkers	1,444	1.27 (1.21, 1.33)	< 0.001	
Non-drinkers	1,336	1.40 (1.31, 1.50)	< 0.001	
**Physical activity**				0.127
Inactivity	1,557	1.28 (1.22, 1.35)	< 0.001	
activity	1,223	1.36 (1.29, 1.45)	< 0.001	
**BMI**				0.655
Normal	415	1.33 (1.16, 1.52)	< 0.001	
Overweight	734	1.17 (1.09, 1.25)	< 0.001	
Obese	1,631	1.14 (1.08, 1.20)	< 0.001	
**Hypertension**				0.191
Yes	1,348	1.34 (1.25, 1.44)	< 0.001	
No	1,432	1.27 (1.21, 1.33)	< 0.001	
**Diabetes**				< 0.001
Yes	746	1.53 (1.34, 1.76)	< 0.001	
No	1,937	1.22 (1.17, 1.27)	< 0.001	
Borderline	97	1.36 (0.998, 1.93)	0.0639	
**Heart failure**				0.105
Yes	176	1.12 (0.934, 1.39)	0.267	
No	2,604	1.32 (1.27, 1.38)	< 0.001	
**Stroke**				0.445
Yes	178	1.45 (1.16, 1.89)	0.00245	
No	2,602	1.32 (1.27, 1.38)	< 0.001	
**Cancer**				0.253
Yes	316	1.42 (1.25, 1.64)	< 0.001	
No	2,464	1.31 (1.26, 1.37)	< 0.001	

PIR, poverty income ratio; BMI, body mass index; CMI, cardiometabolic index.

## 4 Discussion

According to our research, there is a positive correlation between biological aging and CMI. Furthermore, the link persisted even after controlling for other variables, suggesting that CMI was a detrimental element in the biological aging process. A non-linear relationship was identified through dose-response analysis. The inflection points was 1.10 according to threshold effect analysis. This finding can inform more accurate and effective prevention and treatment strategies for biological aging.

CMI is a novel anthropometric measure that shows a strong relationship to metabolic syndrome (Wakabayashi, 2022; Tamini et al., 2024). Numerous studies have shown that CMI is associated with various systemic diseases, highlighting its correlation with worse prognoses. Our results were in alignment with the previous research, which has demonstrated a positive association between biological aging and CMI. Metabolic syndrome is known as a group of risk factors for diabetes and cardiovascular diseases with a pathophysiology closely related to aging (Roddy, 2021; Li et al., 2024b; Oya et al., 2024). Nevertheless, no previous research has examined the relationships between CMI and biological aging. Numerous other anthropometric and metabolic markers, including BMI, triglyceride glucose (TyG) index, WHtR, and visceral adiposity index (VAI), have all been shown to be positively correlated with biological aging. According to a meta-analysis, the epigenetic age of the heavier twins in a BMI-discordant monozygotic twin pair (ΔBMI > 3 kg/m^2^) was 5.2 months older than that of their lighter cotwin (Lundgren et al., 2022). A higher BMI z-score was substantially linked to a faster speed of aging as measured by DunedinPoAm (*b* = 0.0017 adjusting for all covariates). In the relationship between obesity and aging has grown as higher BMI across the lifespan has been linked to early onset of age-related illnesses and mortality (Etzel et al., 2022). In middle-aged and older populations, Qiu et al. (2024) clarified a non-linear connection between the TyG index and the α-Klotho protein (the serum anti-aging protein). When the TyG indices were less than 9.7, no discernible association was seen. Nonetheless, for every unit rise in TyG index over 9.738 there was a corresponding increase in klotho levels of 106.44 pg/ml (Qiu et al., 2024). Additionally, every 0.1 unit rise in WHtR was inversely correlated with the Successful Aging Index (SAI), lowering SAI by nearly 0.5 units (Koloverou et al., 2020). Every additional unit increase in VAI was correlated with a 0.312-year increase in PhenoAgeAccel. Among cancer patients, this positive correlation was more statistically significant. Furthermore, a segmented correlation was observed between VAI and PhenoAgeAccel, with a turning point identified at 10.543 (Xu C. et al., 2024). Additionally, a saturation effect was demonstrated by a nonlinear association between the serum anti-aging protein klotho concentrations and the VAI score. It showed no discernible link when VAI was larger than 3.21, but they were negatively connected when VAI was less than 3.21 (Cui et al., 2023). In the current study, we introduced CMI as a novel predictor of biological aging. To date, this is the first study to evaluate the prognostic value of CMI as a metabolism-related index that is easy to obtain in the context of biological aging. However, additional research is necessary to validate the use of CMI in public health assessments of various specialized populations.

Although CMI is highly related to biological aging as elucidated by our study, the underlying biological mechanisms driving these associations are not fully deciphered. Chronic inflammation and reactive oxygen species (ROS) are thought to be significant factors in the progression of biological aging, which may explain the positive association between CMI and biological aging. The activation of the cyclic GMP-AMP synthase (cGAS)/stimulator of interferon genes (STING) pathway by mitochondria-derived cytosolic DNA (mt-DNA) has been found to produce inflammation factors. Previous studies highlight the crucial role that cytosolic mtDNA-induced cGAS-STING activation plays in the pathophysiology of obesity (Elzinga et al., 2023; Kim et al., 2023; Ma et al., 2023). Microglias exhibit cGAS activity in response to cytosolic DNA released from disrupted mitochondria, indicating a method by which cGAS-STING signaling is activated in the aging brain. Single-nucleus RNA-sequencing analysis of microglia in a cGAS gain-of-function mouse model demonstrates that engagement of cGAS in microglia is sufficient to direct aging-associated transcriptional states leading to bystander cell inflammation (Paul et al., 2021; Gulen et al., 2023; Jiménez-Loygorri et al., 2024). Furthermore, the positive energy balance typical of obesity worsens the excess deposition of ectopic fat with aging. Increased inflammatory cell infiltration and altered chemokine expression, including increased TNF-α and IL-6, are seen in visceral adipose tissue (Colleluori and Villareal, 2021). The increased adipose tissue inflammation with obesity and aging establishes the typical low-grade chronic inflammation observed in older adults (Villareal, 2023). These demonstrate the role of the inflammation in the aging and obesity. ROS as a physiologically significant cause of ribotoxic stress response activation and translational abnormalities (Lennicke and Cochemé, 2021). A significant fraction of the metabolic stress signals responsible for undesirable metabolic maladaptation in obesity and aging stem from damaged ribosomes (Shields et al., 2021; Hajam et al., 2022). ROS-induced ribosome impairment underlies ZAKα-mediated metabolic decline in obesity and aging (Snieckute et al., 2023). Excess calories raise the production of ROS, which harms the mitochondria, endoplasmic reticulum, and nucleus. ROS-induced DNA damage upregulates the cell cycle arrest-related proteins p16 and p21, which causes chromatin rearrangement, cellular senescence, and the release of proinflammatory mediators (Tam et al., 2020). As mentioned, the persistence of DNA damage is a common biological process linking aging and obesity. It is hypothesized that excess leptin synthesis, inflammation, and ROS cause adipose tissue to accumulate DNA damage, which then accumulates mutations in DNA repair genes. Senescence is further induced by the inadequate ability of DNA repair proteins to repair damaged DNA. Therefore, obesity speeds up the aging process by adding to the damage to DNA that comes with aging (Kasper et al., 2022; Kudabayeva et al., 2022; Chowdhury et al., 2023). More investigation is necessary because the precise molecular pathways are not fully understood.

There are various useful implications for this study. It has been discovered that a higher CMI significantly speeds up biological aging. Because the NHANES dataset, on which this study was based, used a fully random sampling procedure, our findings are guaranteed to be representative of the total population. People with high CMI may require additional interventions, such as nutrition, physical activity, and potentially medication-assisted dyslipidemia treatment, to slow down the aging process.

## 5 Study limitations

Firstly, despite adjusting for several confounders, unmeasured or residual confounding cannot be fully excluded. Secondly, treatment factors such oral antidiabetic drugs that could affect CMI were not taken into account. Thirdly, it should be mentioned that participant questionnaires were used to diagnose the study’s cases of hypertension, diabetes, heart failure, cancer, and stroke. This could introduce recollection bias and compromise the study’s ability to make accurate diagnoses. Finally, a number of blood biomarkers were used to determine PA. However, these biomarkers may not correctly reflect other measures of biological aging, such as DNA methylation, telomere length.

## 6 Conclusion

After adjusting for potential confounders, our research demonstrated a positive correlation between CMI and biological aging. CMI be integrated into routine health screenings or aging assessments by healthcare professionals. Further cohort studies or randomized controlled trials are desperately needed to validate this result to provide more effective prevention and treatment strategies for biological aging in the future.

## Data Availability

The original contributions presented in this study are included in this article/supplementary material, further inquiries can be directed to the corresponding author.
